# The Use of the Central Vein Sign in the Diagnosis of Multiple Sclerosis: A Systematic Review and Meta-analysis

**DOI:** 10.3390/diagnostics10121025

**Published:** 2020-11-29

**Authors:** Marco Castellaro, Agnese Tamanti, Anna Isabella Pisani, Francesca Benedetta Pizzini, Francesco Crescenzo, Massimiliano Calabrese

**Affiliations:** 1 Department of Neurosciences, Biomedicine and Movement Sciences, University of Verona, 37134 Verona, Italy; agnese.tamanti@univr.it (A.T.); annaisabella.pisani@univr.it (A.I.P.); francescocrescenzo89@gmail.com (F.C.); massimiliano.calabrese@univr.it (M.C.); 2 Department of Diagnostics and Public Health, University of Verona, 37134 Verona, Italy; francescabenedetta.pizzini@univr.it

**Keywords:** multiple sclerosis, MS, central vein sign, perivenular, lesion, imaging, MRI, biomarker

## Abstract

***Background***: The central vein sign (CVS) is a radiological feature proposed as a multiple sclerosis (MS) imaging biomarker able to accurately differentiate MS from other white matter diseases of the central nervous system. In this work, we evaluated the pooled proportion of the CVS in brain MS lesions and to estimate the diagnostic performance of CVS to perform a diagnosis of MS and propose an optimal cut-off value. ***Methods***: A systematic search was performed on publicly available databases (PUBMED/MEDLINE and Web of Science) up to 24 August 2020. Analysis of the proportion of white matter MS lesions with a central vein was performed using bivariate random-effect models. A meta-regression analysis was performed and the impact of using particular sequences (such as 3D echo-planar imaging) and post-processing techniques (such as FLAIR*) was investigated. Pooled sensibility and specificity were estimated using bivariate models and meta-regression was performed to address heterogeneity. Inclusion and publication bias were assessed using asymmetry tests and a funnel plot. A hierarchical summary receiver operating curve (HSROC) was used to estimate the summary accuracy in diagnostic performance. The Youden index was employed to estimate the optimal cut-off value using individual patient data. ***Results:*** The pooled proportion of lesions showing a CVS in the MS population was 73%. The use of the CVS showed a remarkable diagnostic performance in MS cases, providing a pooled specificity of 92% and a sensitivity of 95%. The optimal cut-off value obtained from the individual patient data pooled together was 40% with excellent accuracy calculated by the area under the ROC (0.946). The 3D-EPI sequences showed both a higher pooled proportion compared to other sequences and explained heterogeneity in the meta-regression analysis of diagnostic performances. The 1.5 Tesla (T) scanners showed a lower (58%) proportion of MS lesions with a CVS compared to both 3T (74%) and 7T (82%). ***Conclusions:*** The meta-analysis we have performed shows that the use of the CVS in differentiating MS from other mimicking diseases is encouraged; moreover, the use of dedicated sequences such as 3D-EPI and the high MRI field is beneficial.

## 1. Introduction

Multiple sclerosis (MS) is one of the most common autoimmune disorders of the central nervous system (CNS), characterized by diffuse inflammatory/demyelinating and neurodegenerative alterations [1]. Magnetic resonance imaging (MRI) is one of the most powerful technological aids supporting MS diagnosis which can be used to demonstrate the distinctive dissemination in space and time of the disease. Over time, the diagnostic criteria, also implementing MRI in the diagnostic process, have increased their sensitivity, allowing an even earlier MS diagnosis, after a first/isolated event suggestive of acute CNS demyelination [2]. However, their specificity remains limited, especially in the presence of atypical history, and this could result in misdiagnosis and initiation/delay of inappropriate/approved disease-modifying treatment [3]. Brain MRI findings in conventional T2-weighted sequences in patients with different morbidities, such as vasculopathies, migraine and non-MS CNS demyelinating or autoimmune disorders, may be mistakenly suggestive of MS lesions [4]. Therefore, more MS-specific MRI criteria to rule out MS-mimicking disorders are needed.

The central vein sign (CVS), a recent radiological feature developed initially in ultra-high field MRI studies [5,6], relying on the pathological specificity of perivenular distribution of MS lesions [7], has been proposed as an MS imaging biomarker able to accurately differentiate MS from other white matter (WM) diseases of the CNS.

Although a definition of the CVS has been provided by an expert consensus, briefly as a distinct vein centrally located in a clearly visible MS lesion > 3 mm in diameter irrespective of its location in supratentorial brain white matter [8], and criteria based on both *proportion lesion threshold* [9,10] or *lesion number threshold* [11,12] have been proposed to discriminate MS from its MRI mimics, the lack of both a standardized imaging acquisition protocol and an accurate (and routinely suitable) CVS evaluation approach in suspected MS cases currently still prevent the applicability of this potential biomarker.

**Figure 1:**
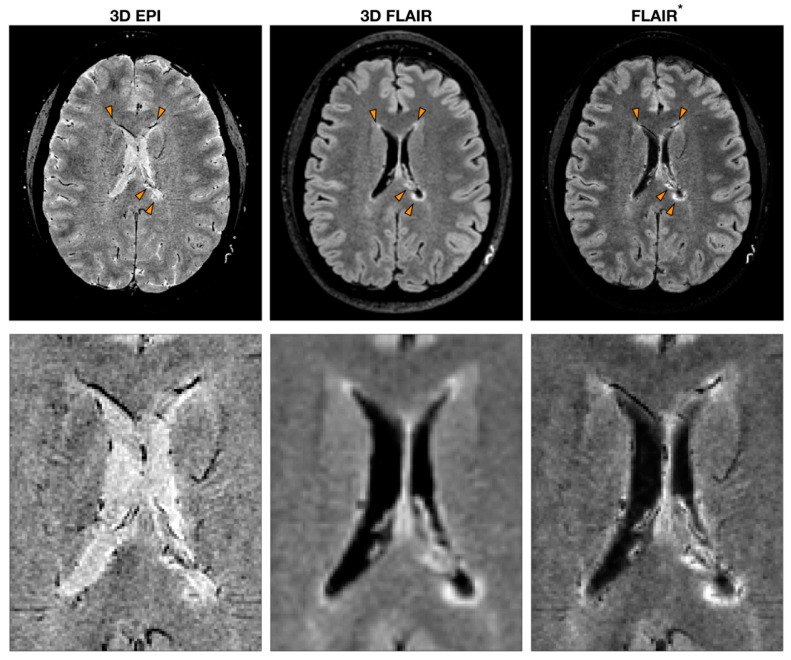
Example of multiple sclerosis (MS) patient MRI images obtained with sequences to detect the central vein sign (CVS). T2*-weighted 3D echo-planar imaging (EPI), 3D fluid-attenuated inversion recovery (FLAIR) and FLAIR*, a post-processing technique that combines the EPI sequence [or more generally a T2*w sequence (used to detect CVS)] and FLAIR. The orange arrowheads indicate MS lesions with the characteristic CVS. A zoomed-in version of the same image is reported to highlight the CVS feature for each modality.

Imaging of a central vein in MS lesions can be achieved by exploiting the ability of susceptibility (T2*-“T2-star”)-based MRI sequences to highlight in vivo paramagnetic properties of deoxyhemoglobin in venous blood flow [13]. By combining both magnitude and phase images acquired using T2*-weighted (T2*w) gradient echo (GRE) pulse sequences, it is possible to exploit susceptibility-weighted imaging (SWI) that can reveal the presence of blood vessels in MS lesions [14,15]. Even though the core protocol always includes a GRE sequence, a great variety of MRI parameters can be utilized to obtain SWI [14]. For instance, some studies introduced the use of echo-planar imaging (EPI) that allows for the retrieval of high-resolution images while reducing the scan time [16]. Further heterogeneity in the studies is introduced by the post-processing implementation. The first processing step required to compute SWI is the removal of artifacts from phase images. Several different phase processing approaches have been tested where high-pass filters are applied directly to phase images or following phase unwrapping [17]. Besides obtaining SWI from the combination of both magnitude and phase from the same GRE acquisition, another strategy consists of multiplying a T2*-weighted sequence with the fluid-attenuated inversion recovery (FLAIR) sequence to obtain a FLAIR* image [18]. This approach combines the sensibility of FLAIR in detecting WM lesions and the ability of susceptibility-based imaging to highlight the presence of the vein (Figure 1).

In this meta-analytic study, we aim to systematically review the proportion of MS white matter lesions which show the CVS (MSL Vein+) and estimate the performance diagnostic value, in terms of specificity, sensitivity and accuracy, in discriminating MS from its common radiological differential diagnosis, aiming to endorse its usefulness in clinical practice. Moreover, we further investigate the impact of using different field strengths, specific sequences (i.e., 3D echo-planar imaging) and post-processing techniques (i.e., SWI, FLAIR*).

## 2. Materials and Methods

The systematic review and meta-analysis were performed following the Preferred Reporting Items for a Systematic Review and Meta-analysis of Diagnostic Test Accuracy (PRISMA-DTA) statement [19,20]. The methodological framework used in this review is consistent with a previously published review and meta-analysis on the topic [21,22].

### 2.1. Literature Search: Databases and Search Phrase

Systematic research over two major databases was conducted following a recent systematic review and meta-analysis guideline [23]. PUBMED/MEDLINE and Web of Science were selected to look for articles using the CSV criteria to strengthen the MS diagnosis capability. The search string used in both databases was: (((“multiple sclerosis”) AND ((“central vein”) OR ((“vein”) AND (“lesion”)) OR (“perivenular”))). The literature research included only papers written in English and published up to 24 August 2020. The management of the literature research was conducted with Zotero (v 5.0.90).

### 2.2. Eligibility Criteria

Among studies retrieved from searched databases, we selected only published peer-reviewed studies that met all the following criteria: studies including patients with MS; studies evaluating the CSV. Furthermore, we included studies that provided either the prevalence of the CSV in white matter lesions or true positives (TPs), false negatives (FNs), true negatives (TNs) and false positives (FPs) in diagnosing MS using the CVS.

We excluded from the review and from the meta-analysis any paper that met any of the following criteria: conference proceedings; animal-based studies; reviews; case reports and papers with fewer than five cases; editorial notes, surveys and guidelines.

### 2.3. Paper Summary and Data Extraction

The prevalence of the CVS in MS lesions and its diagnostic performance in differentiating MS from other diseases were separately summarized for each article included in the review. The CVS has been recently defined by the guidelines of the North American Imaging in Multiple Sclerosis Cooperative (NAIMS Cooperative) [8]. Accordingly, in this review, a central vein was defined as exhibiting the following properties in T2*-weighted images:

- Appears as a thin hypointense line or small hypointense dot;
- Has a small apparent diameter;
- Runs partially or entirely through the lesion;
- Is positioned centrally in the lesion, regardless of the lesion’s shape.

Some studies (i.e., those published before the NAIMS Cooperative consensus paper) were included in this review, although the exclusion criteria for scoring the CVS according to the NAIMS Cooperative consensus were fulfilled (i.e., confluent lesion evaluation).

Diagnostic performances in terms of TP, FN, TN and FP for each study were recorded when available or were derived if not explicitly stated in the paper. Moreover, for each study, we also reported: study design (prospective/retrospective), the number of patients in the MS cohort and in the control group; sub-division between MS phenotypes; differential diagnosis; age (mean and interval) of both the MS and the control group; magnetic field strength; sequence used in the evaluation of the CVS; use of contrast agent; the number of readers and their background and blindness to the reference standard; institution and type of study (mono-/multi-centric); cut-off for the prevalence of the CVS in MS lesions and rule used (if multiple thresholds/criteria/rules were used, only the overall best diagnostic performance, in terms of the maximum sum of specificity and sensitivity, was retained).

### 2.4. Statistical Analysis

Estimations of pooled incidence of the central vein sign in T2*-based MRI acquisition in patients with MS were carried out using a random-effects meta-analysis weighted with the inverse variance supporting DerSimonian–Laird’s method [24]. Heterogeneity between studies was quantified by the Higgins inconsistency test through the I^2^ index which is not directly influenced by the number of the studies included in the analysis. The I^2^ index lies between 0% and 100%. A value of 0% implies no observed heterogeneity, while larger values indicate increasing heterogeneity (25, 50 and 75% indicate low, medium and high heterogeneity, respectively). Publication bias was detected by funnel plots and the asymmetry was assessed by Egger’s test. To explain the effects of study heterogeneity, a meta-regression analysis was performed using the study design (prospective study vs. others), post-processing image technique (FLAIR* vs. others), MRI sequence ( 3D-EPI vs. others), reader (radiologist vs. other), patient age (age median ≤ average age weighted on patient number vs. age median > age weighted on patient number), use of the contrast agent before the acquisition of the T2*-weighted sequence (used vs. not used) and blindness to the reference standard as covariates. Sub-group analysis was performed to test the single effect of the scanner magnetic strength (1.5 Tesla (T) vs. 3T vs. 7T), type of post-processing employed (FLAIR* vs. others) and MRI sequence employed (3D-EPI vs. others). The proportional meta-analysis conducted on both the complete set of studies included in this work and separately for each sub-group was preceded by an influence analysis to identify outliers in each set or subset [25].

The bivariate random-effects model was made in order to calculate the pooled sensitivity and specificity for the diagnostic accuracy of the central vein sign for differentiating MS from other common differential diagnoses. The estimated sensitivity and specificity and the credible intervals for each study were reported in a forest plot. The hierarchical summary receiver operating characteristic (HSROC) curve with 95% confidence and prediction regions was plotted. The extent of heterogeneity was assessed using the Cochran Q-test, the Higgins I^2^ test and the Spearman correlation coefficient (rho) between sensitivity and false positive rate. Possible publication bias was evaluated by Deeks’ funnel plot and assessed with a regression test on the diagnostic odds ratio. A *p*-value < 0.05 was considered as indicative of statistical significance.

A bivariate meta-regression model was applied to evaluate the effects of study heterogeneity using the following seven covariates: study design (prospective study vs. others), post-processing technique (FLAIR* vs. others), MRI sequence (3D-EPI vs. others), reader (radiologist vs. other), patient age (age median ≤ average age weighted on patient number vs. age median > age weighted on patient number), use of the contrast agent before the acquisition of the T2*-weighted sequence (used vs. not used) and blindness to the reference standard. Finally, subgroup analysis was performed using the proportion of lesions with the central vein sign as a cut-off value on those studies providing individual patient data. The individual data were extracted from the text of the articles or estimated from the plots indicating the proportion of lesions with the central vein sign using Web Plot Digitizer v. 4.3 (https://automeris.io/WebPlotDigitizer/) when data were not reported. The optimal threshold in ROC analysis was defined using the Youden index method [26]. Specifically, the Youden index was applied to estimate the optimal cut-off value for the proportion of lesions with the central vein sign and the corresponding sensitivity and specificity (95% CI). The Youden index is defined as sensitivity + specificity -1, with it having a minimum value of −1 and a maximum value of + 1, with a value of + 1 indicating the optimal value for an algorithm. Statistical analysis was performed using the “metafor”, “mada” and “meta4diag” packages in R v. 3.5 and the “metandi” and “midas” modules in STATA 16.1.

## 3. Results

### 3.1. Literature Review

**Figure 2:**
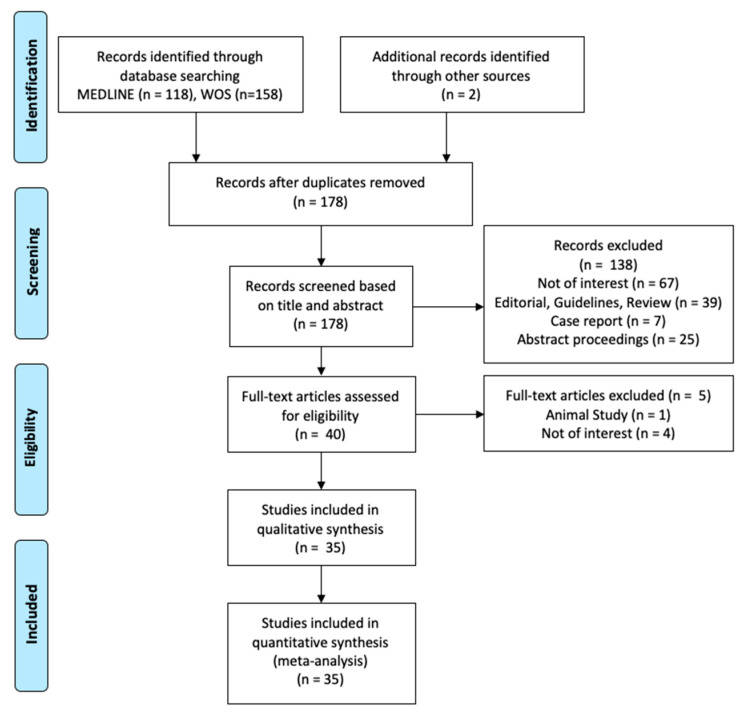
Preferred Reporting Items for a Systematic Review and Meta-analysis of Diagnostic Test Accuracy (PRISMA-DTA) flow diagram to select studies to be included in the subsequent meta-analysis.

A total of 278 studies were screened according to the Preferred Reporting Items for a Systematic Review and Meta-analysis of Diagnostic Test Accuracy (PRISMA-DTA) (Figure 2). For subsequent qualitative and quantitative analysis, a total of 35 studies were considered eligible. A total of 1047 patients, including clinically isolated syndrome (CIS; *n* = 256) or definite multiple sclerosis (MS; *n* = 791), were included in the analysis.

### 3.2. Features of the Eligible Studies

Eleven studies used a prospective design [9,27,28,29,30,31,32,33,34,35,36], while ten reported a retrospective one [11,12,37,38,39,40,41,42,43,44]. The remaining fourteen did not mention the design type or the studied cohort was partially collected prospectively and retrospectively [10,38,45,46,47,48,49,50,51,52,53,54,55,56]. The mean number ± standard deviation of MS patients included in each study was 45.3 ± 70.78 (range: 5–323).

Five studies used a 1.5T scanner [27,38,39,42,43], twenty-one a 3T scanner [11,12,28,29,30,31,32,33,35,36,37,40,46,47,48,51,52,53,54,55,57] and six a 7T scanner [10,34,41,45,50,58]. The remaining studies used images acquired at multiple field strengths: one study used either 1.5T or 3T but did not provide the proportion of CVS lesions for each scanner and thus was excluded from the sub-group analysis based on the scanner field strength [9]; two studies used both 3T and 7T scanners, however, in our meta-analysis, only the results from the highest field strength were reported as they provided the best performance [49,56].

Even though all the eligible studies included a susceptibility-based sequence (either susceptibility-weighted images, SWI; T2*-weighted gradient echo, T2*w; or T2*-weighted 3D echo-planar imaging, 3D-EPI), several protocols and processing techniques have been reported. Of the three studies detecting the CVS in T2*w images [45,50,58], one selected contrast-enhancing lesions using post-contrast images [45]. Of the fifteen studies using SWI [27,30,32,35,37,38,39,40,41,42,43,47,48,52,54] to identify the central vein sign, one added to the MRI protocol post-contrast sequences to select contrast-enhancing lesions [39], eight added a FLAIR sequence [30,35,40,42,47,48,52,54], one added a conventional T2-weighted acquisitions [32] and four added both FLAIR and conventional T2-weighted acquisitions [27,37,38,43], to delineate white matter lesions. Six studies employed a T2*w EPI sequence [10,11,33,34,46,57] and eight studies acquired both EPI and FLAIR sequences [9,12,28,29,36,49,53] that were combined to obtain FLAIR* images in seven of these studies [9,12,28,29,31,36,53]. Of the remaining studies, one retrieved FLAIR* images from the post-processing of a 3T FLAIR and the phase of a 7T SWI sequence [56], one used multiple susceptibility-weighted qualitative and quantitative images (SWI and R2*, respectively) [55] and one was a multicentric study including a heterogeneous set of MRI sequences [51]. In ten of the eligible studies, the susceptibility-based sequence was acquired during or after the injection of the contrast agent [9,12,28,30,31,39,40,47,53].

### 3.3. MS Lesions with Central Vein Pooled Proportion

**Figure 3:**
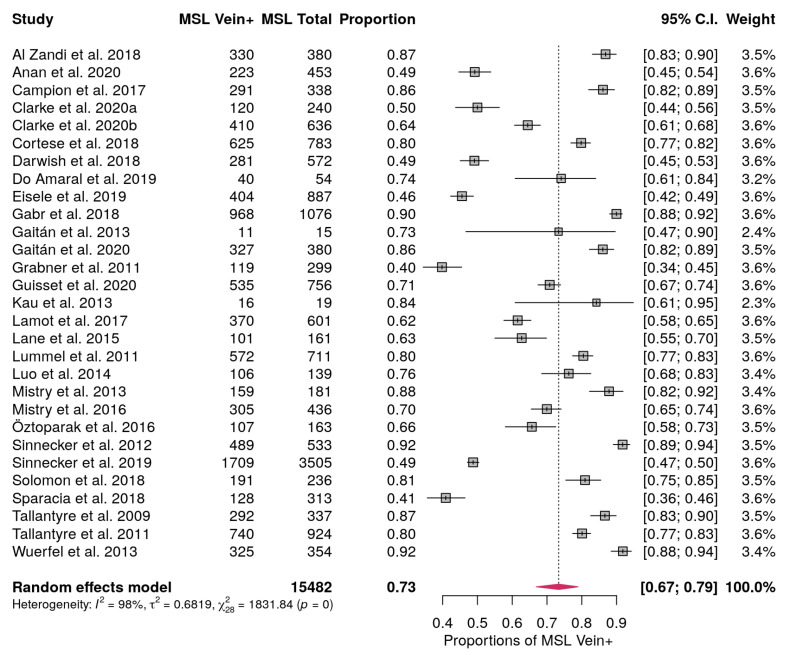
Forest plot showing the proportion of MS lesions with central vein sign (MSL Vein+) and the weight in the Random effect model for each study considered. Proportions are reported with estimated 95% confidence intervals (C.I.). In bold are reported the total number of MS lesions, the pooled proportions and the pooled C.I. and the total weights.

Among the studies included, twenty-nine provided information on the proportion of MS lesions with the central vein sign [10,11,12,27,28,29,30,32,33,34,35,36,37,38,39,40,42,43,47,48,49,50,51,52,54,55,56,58,59]. The proportion of lesions with a central vein sign ranged from 0.4 up to 0.92. The pooled proportion of central vein signs in MS lesions considering all the studies included was 0.73 (95% CI, 0.67–0.79 (Figure 3). Influence analysis to detect outliers did not find any study that largely impacted on the pooled proportion. The Higgins inconsistency test showed high heterogeneity (I^2^ = 98%). The meta-regression carried out to describe this heterogeneity did not find any statistical significance among the modeled covariates. The publication bias assessment performed with the funnel plot showed no asymmetry (Supplementary Figure S1) and Egger’s test showed no statistically significant asymmetry (*p* = 0.074).

Regarding the effect of the scanner magnetic strength (1.5T vs. 3T vs. 7T), we did not find any study largely driving any subgroup. Moreover, the pooled proportion of central veins obtained with 1.5T MRI scanners was 0.58 (95% CI, 0.47–0.68). It was statistically different compared to the proportion obtained with both 3T (0.74 - 95% CI, 0.66–0.81, *p* = 0.011) and 7T (0.82 - 95% CI, 0.67–0.91, *p* = 0.011) scanners (Supplementary Figure S2). There was no statistically significant difference in the pooled proportion between 3T and 7T (*p* = 0.32).

The type of the post-processing employed (FLAIR* vs. others) did not show any influential study nor any statistically significant difference in the pooled proportion, that was 0.77 (95% CI, 0.61–0.87) when employing the FLAIR* and 0.74 (95% CI, 0.66–0.80) when it was not used (Supplementary Figure S3).

The MRI sequence used in the acquisition (3D-EPI vs. others) showed a study influencing the pooled proportion in the 3D-EPI sub-group [48], that was excluded from further sub-group analysis. After removing this outlying study, the proportion of lesions showing a central vein when using 3D-EPI was 0.82 (95% CI, 0.76–0.87) and it was statistically significantly higher (*p* = 0.018) compared to the use of other sequences (0.71 - 95% CI, 0.62–0.78, Supplementary Figure S4).

### 3.4. MS Diagnostic Accuracy Measured by Pooled Sensitivity and Specificity

**Figure 4:**
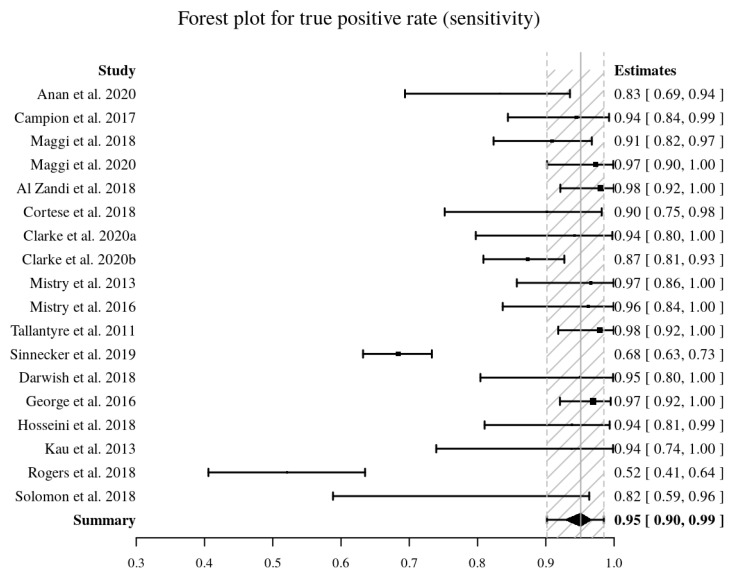
Forest plot of pooled sensitivity (in bold) of the central vein sign in T2*-weighted images for differentiating MS from other mimicking diseases. Sensitivity values are reported with estimated 95% confidence intervals (CIs) for each study included.

**Figure 5:**
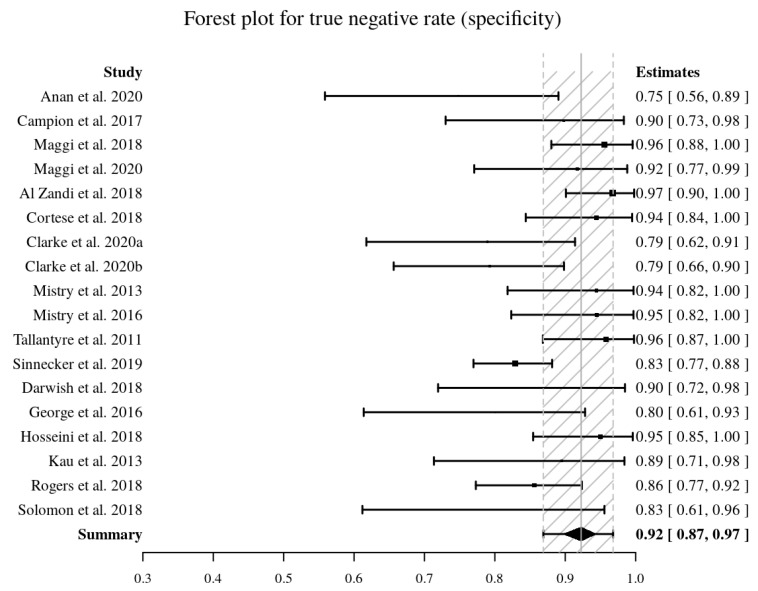
Forest plot of pooled specificity (in bold) of the central vein sign in T2*-weighted images for differentiating MS from other mimicking diseases. Specificity values are reported with estimated 95% confidence intervals (CIs) for each study included.

**Figure 6:**
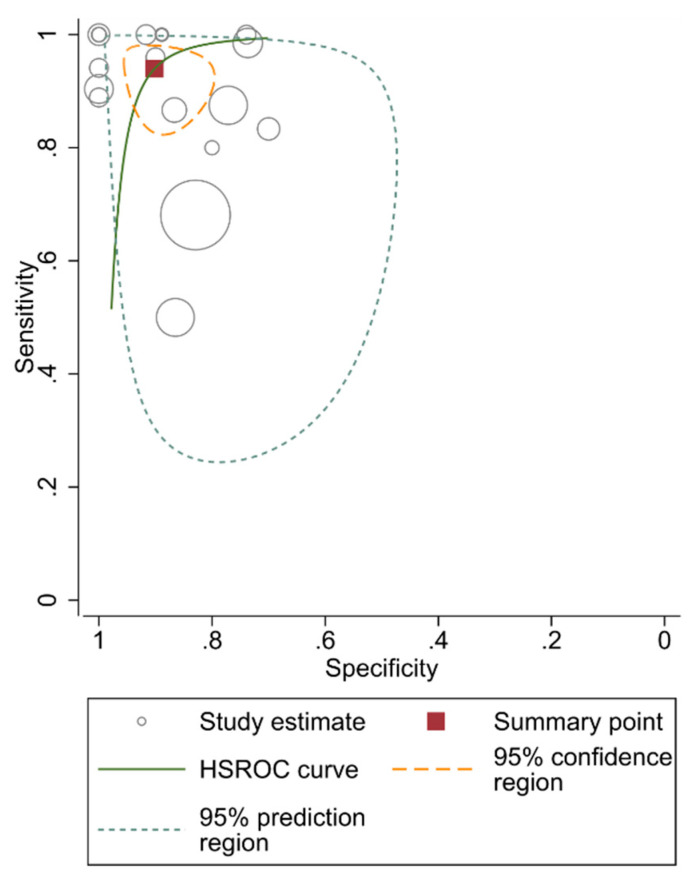
Hierarchical summary receiver operating characteristic (HSROC) curve of the diagnostic performance of the central vein sign in T2*-based images for differentiating MS from other mimicking diseases.

Eighteen studies [9,10,11,12,27,28,32,33,34,35,41,44,47,48,51,52,53] provided the data to perform a pooled diagnostic accuracy meta-analysis. The pooled sensitivity and pooled specificity were 95% (95% CI, 90–99%) and 92% (95% CI, 87–97%), respectively (Figure 4, Figure 5). The area under the HSROC curve was 0.98 (95% CI, 0.96–0.99%) (Figure 6). Both the Q-test (Q = 18.915, *p* = 0.333) and the Higgins I^2^ (10.12) suggested low heterogeneity across the studies. Both the forest plots and the Spearman correlation (rho = −0.18, 95% CI, −0.595–0.316) coefficient revealed no evidence of a threshold effect. Publication bias was detected by Deeks’ funnel plot (*p* < 0.001, Supplementary Figure S5).

In the meta-regression, among the covariates, only the MRI sequence (*p* = 0.04) significantly affected the heterogeneity. Other covariates, including study design (*p* = 0.24), reader blindness to the reference standard (*p* = 0.48), age (*p* = 0.06), reader (*p* = 0.48) and post-processing technique (*p* = 0.20), did not show statistical differences.

### 3.5. Diagnostic Performance using Distinct Patient Data

**Figure 7:**
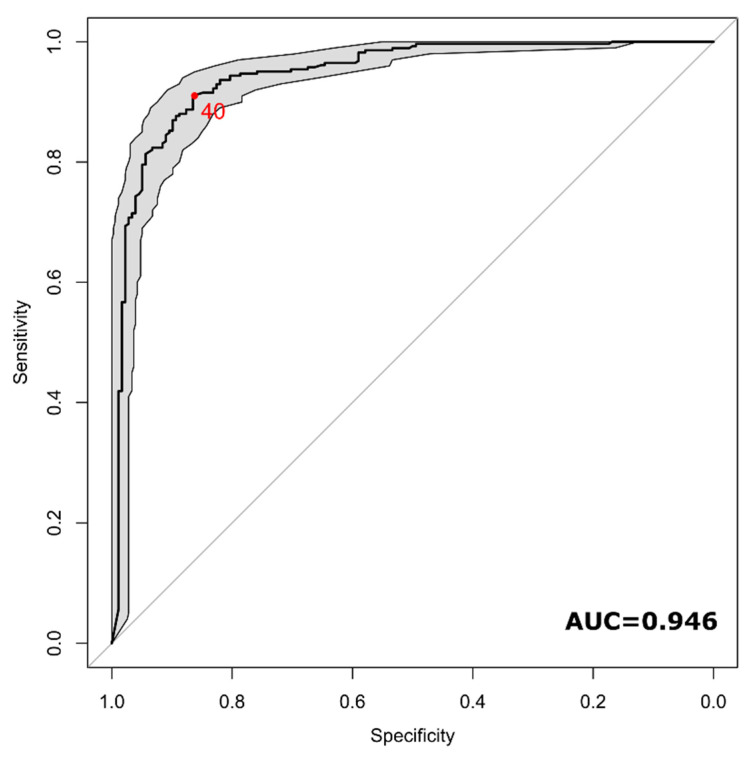
ROC curve of the diagnostic performance of central vein sign in T2*-based images for differentiating MS from other mimicking diseases at an individual level. The red dot indicates the optimal cut-off identified (40%).

Fifteen studies reported the single patient percentage of lesions with a central vein [9,10,11,27,28,32,34,36,41,46,48,50,52,57,58]. The number of considered MS patients was 284, while 178 were patients with other diseases or healthy controls (58% of MS patients). The cut-off values among the considered distinct patients ranged from 0 to 100 with a median value of 54%. The area under the ROC curve of the proportion of lesions with the central vein sign for the diagnosis of MS was 0.946 (95% CI, 0.924–0.967) (Figure 7). The optimal cut-off value identified by the Youden approach was 40%, with a sensitivity of 90% (95% CI, 81–95%) and a specificity of 89% (95% CI, 82%–96%).

## 4. Discussion

Evaluating the role of more specific magnetic resonance imaging (MRI) features of multiple sclerosis (MS) lesions, such as the central vein sign (CVS), is considered a high-priority area for research according to The International Panel on Diagnosis of MS, which produced the 2017 revised diagnostic criteria [60].

The systematic review and meta-analysis we performed aimed to assess the prevalence of the CVS in white matter (WM) MS brain lesions with respect to other WM diseases, as well as to investigate the use of the CVS in the differential diagnosis of MS by suggesting an optimal cut-off to differentiate MS from other conditions with MRI T2 hyperintensities and analyze the diagnostic performance measured with pooled sensitivity and specificity.

In this regard, the pooled proportion analysis revealed a high proportion (73%) of lesions showing a CVS in MS. Moreover, the use of the CVS reported a notable diagnostic performance, providing a pooled specificity of 92% and sensitivity of 95%. The pooled diagnostic performances in differentiating MS from other diseases estimated with the area under the HSROC of 0.98 (95% CI, 0.96–0.99%) revealed an excellent performance in this task. The optimal cut-off value obtained from the single patient data pooled together was 40% with an excellent value of the accuracy calculated by the area under the ROC (0.946). The heterogeneity in the dataset is high and this can be explained by the various features and settings of the eligible studies in terms of sequence, scanner field strength, post-processing technique and control group (both MS mimics and healthy control groups were considered). Nevertheless, the analysis conducted supports the potential role of the CVS in the diagnostic work-up of MS. Among several neuropathological features that characterize MS lesions, despite their different and asynchronous evolutive aspects over time, the vasocentricity is one of the most specific [61], in line with the generally accepted pathogenic theory that the early formation of a demyelinating focal area—an MS lesion—depends on the entry of lymphocytes and other inflammatory cells from the systemic circulation into the central nervous system across the blood–brain barrier [1]. WM lesions in MS, following the parenchymal venous topography (mainly at the level of small and medium veins), are commonly found in periventricular, infratentorial and medullary areas (deep venous system), as well as in juxtacortical regions (superficial venous system) and within the optic nerve (central retinal vein). Therefore, the careful evaluation of WM lesion distribution is certainly helpful in distinguishing MS from its MRI mimics, although some of them can involve the same areas, leading to image misinterpretation [62]. An elevated concentration of deoxyhemoglobin—higher oxygen consumption—and increased venous diameters—increased venous flow—at the inflamed site could be some of the reasons for peculiar vessel evidence by MR venography in MS lesions [63], even though this is still a topic of investigation.

One of the goals of our study was to investigate the optimal cut-off for WM lesions showing the CVS to differentiate between MS and other diseases. The cut-off values for the proportion of lesions with the CVS for the diagnosis of MS at the single-patient level ranged from 0 to 100. We found an optimal threshold of 40%, that has been reported in several single studies [9,10,27,31,33,34,46,48,53], confirming this threshold as an excellent value with a sensitivity of 90% and a specificity of 89%. However, manually counting and visually detecting the presence of the CVS for each lesion is very time consuming, influenced by both intra- and inter-observer variability and prone to error due to repetitiveness and the tiredness of the observer performing the task. Therefore, reliable and robust complete automatic detection and counting are highly desirable. A few attempts have been made based on classical machine learning and novel deep learning methods [64,65] but further studies to validate these promising methods are needed. A portion of the studies included in this review used the FLAIR* approach [18], but the analyses included in this study did not provide any difference in the proportion of detectable CVS-positive lesions when using this approach with respect to others. The limited number of studies employing this method available for this review might have influenced this result, however, this approach might be rarely accessible in a clinical setting since to produce FLAIR* images, processing steps such as coregistration and multiplication, often not implemented directly in MRI scanners, are necessary. Another interesting aspect highlighted by this examination is that both the choice of the sequence as well as the scanner field strength play an important role in the identification of the CVS. As expected, the higher signal-to-noise ratio and contrast-to-noise ratio of high-field MRI scanners do contributes to better delineate and detect the CVS (0.82), however, we found that the use of 3T scanner might be sufficient in this regard (0.74), whereas the use of 1.5T scanners showed a statistically significant reduction in CSV detection (0.58). Among sequences, we found a statistically significant improvement of the CVS proportion detected in MS lesions using a 3D-EPI sequence versus all other sequences (0.82 vs. 0.71) regardless of the field strength. However, this particular sequence is not routinely used, perhaps due to the unavailability of a standard product sequence from some manufacturers. Therefore, a standardization and a larger study on this aspect should be envisaged. Another aspect that should be better investigated is the use of the contrast agent with this particular sequence. A quantitative evaluation of the beneficial use of the contrast agent in the detection of the CVS has not yet been performed, nevertheless, some studies [16,66,67] reported a notable improvement in this regard.

Two meta-analyses of using the CVS in MS were performed recently [21,22]. Our results are in agreement with Suh et al. [21], showing a similar proportion of lesions with the CVS and a slightly lower optimal cut-off value of 40% compared to 45%, even if this does not include the largest European multicenter study on the diagnostic value of the CVS in MS to date [51]. We further provide insight on the use of the 3D-EPI sequence and we also found a significantly different proportion of MS lesions with the CVS by comparing 1.5T and 3T scanners, suggesting that 3T scanners might provide a better performance. A more recent meta-analysis [22] was performed only with 1.5T and 3T and provided a lower proportion of MS lesions with the CVS than our findings, suggesting a threshold of 45% for the differentiation of MS from radiological mimics, however, this study included only a small portion of the articles included in our review.

Nevertheless, this work has some limitations. Regarding publication bias, we verified that the proportional analysis did not show any significant publication bias effect, while the pooled specificity and sensitivity analysis reported a publication bias assessed by Deeks’ funnel plot and the regression test on the diagnostic odds ratio (*p* < 0.001), suggesting that the diagnostic performances could have been overestimated. We have included studies with a control population composed of heterogeneous mimicking disease spectra and also healthy controls that are vulnerable to selection bias. The studies included are even more heterogeneous in terms of the number of subjects (range: 5–323) as well as scanner field strength, sequence and post-processing technique used. Taking together the above reported limitations, special care should be taken when using the CSV in routine clinical practice. 

## 5. Conclusions

Despite the high heterogeneity of the studies included in this meta-analysis, the CVS in differentiating MS lesions from other confounding disease could be used. We do highlight that the minimum scanner field strength needed to better exploit the CVS specificity is 3T, and the T2*-weighted 3D-EPI sequence could be suggested as a preferable image acquisition strategy.

## Supplementary Materials

The following are available online at https://www.mdpi.com/2075-4418/10/12/1025/s1, Supplementary Figure S1: Funnel plot of proportional meta-analysis; Supplementary Figure S2: Forest plot of the sub-group analysis based on the scanner field strength; Supplementary Figure S3: Forest plot of the sub-group analysis based on the post-processing technique; Supplementary Figure S4: Forest plot of the sub-group analysis based on the MRI sequence performed; Supplementary Figure S5: Deeks’ funnel plot of diagnostic performance.
