# Supplementary material for: The Use of the Central Vein Sign in the Diagnosis of Multiple Sclerosis: A Systematic Review and Meta-analysis
Source: Diagnostics (Basel). 2020 Nov 29;10(12):1025. doi: 10.3390/diagnostics10121025 (PMC7760678; doi:10.3390/diagnostics10121025)
Supplement: Supplementary file 1 [file diagnostics-10-01025-s001.pdf]

Supplementary Materials

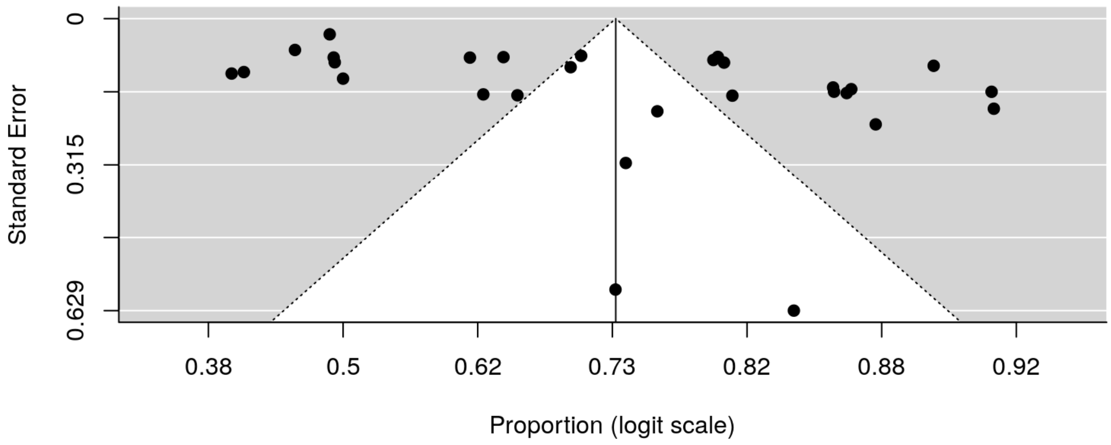

Figure 1. Funnel plot of proportional Meta-Analysis to evaluate the risk of publication bias in assessing the proportion of MS lesion with the central vein sign.

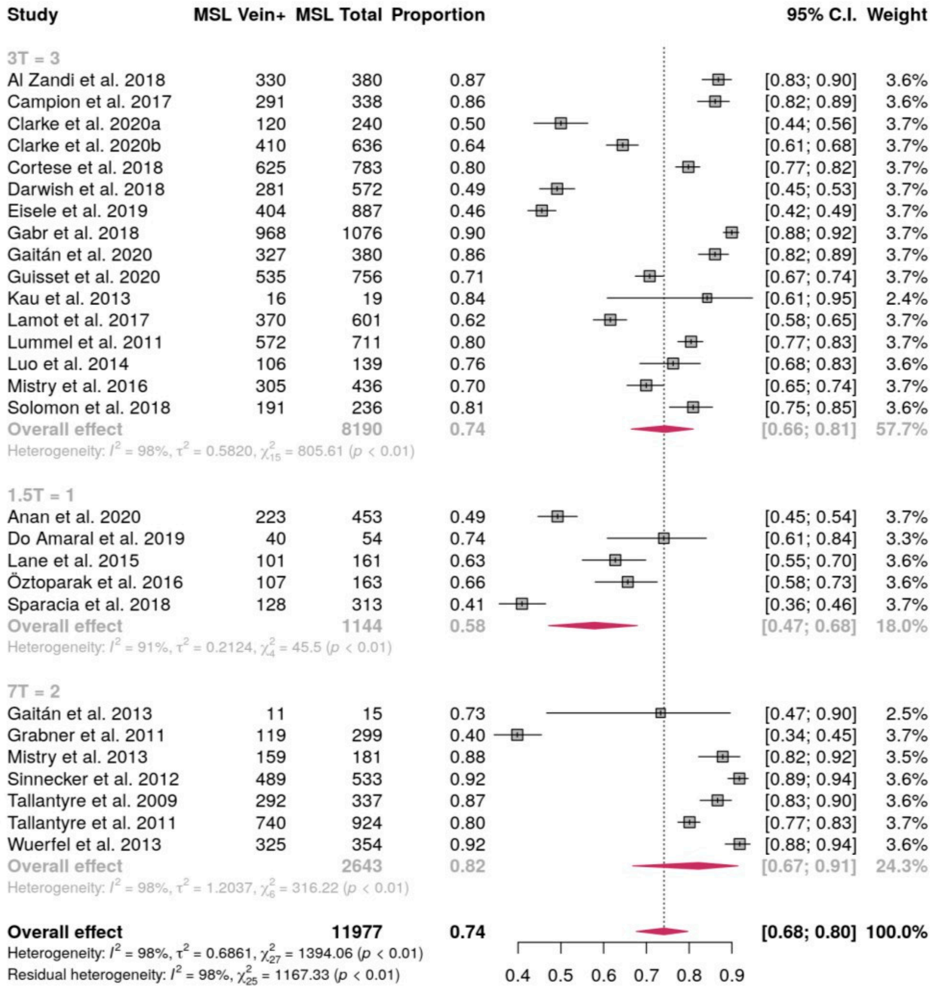

Figure 2. Forest plot of the sub-group analysis based on the scanner field strength (1.5T, 3T or 7T) showing the proportional Meta-Analysis of the pooled proportion of MS lesion with central vein sign (MSL Vein+).

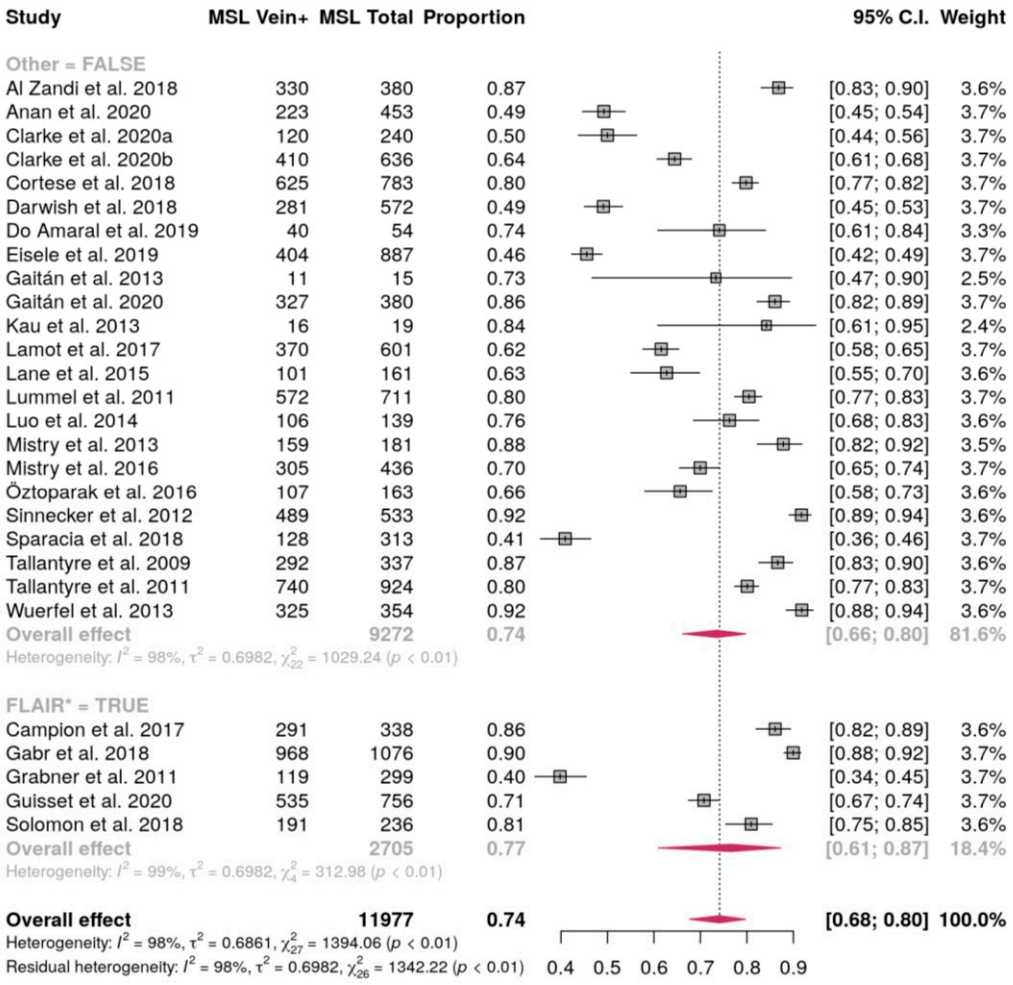

Figure 3. Forest plot of the sub-group analysis based on the post-processing technique used (FLAIR* vs. others) showing the proportional Meta-Analysis of the pooled proportion of MS lesion with central vein sign (MSL Vein+).

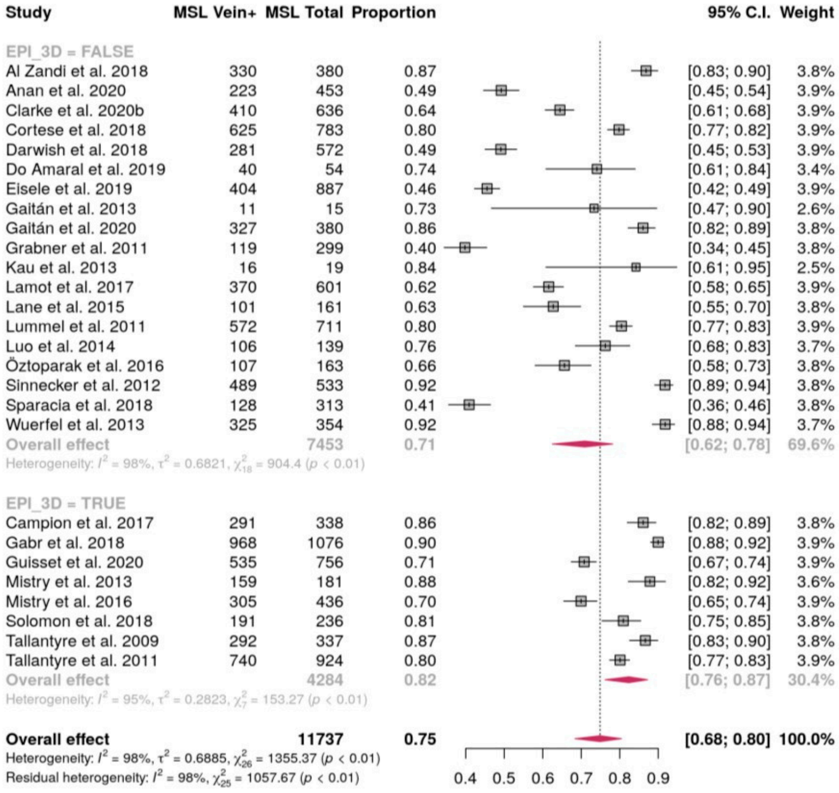

Figure 4. Forest plot of the sub-group analysis based on the MRI sequence performed (3D-EPI vs. others) showing the proportional Meta-Analysis of the pooled proportion of MS lesion with central vein sign (MSL Vein+).

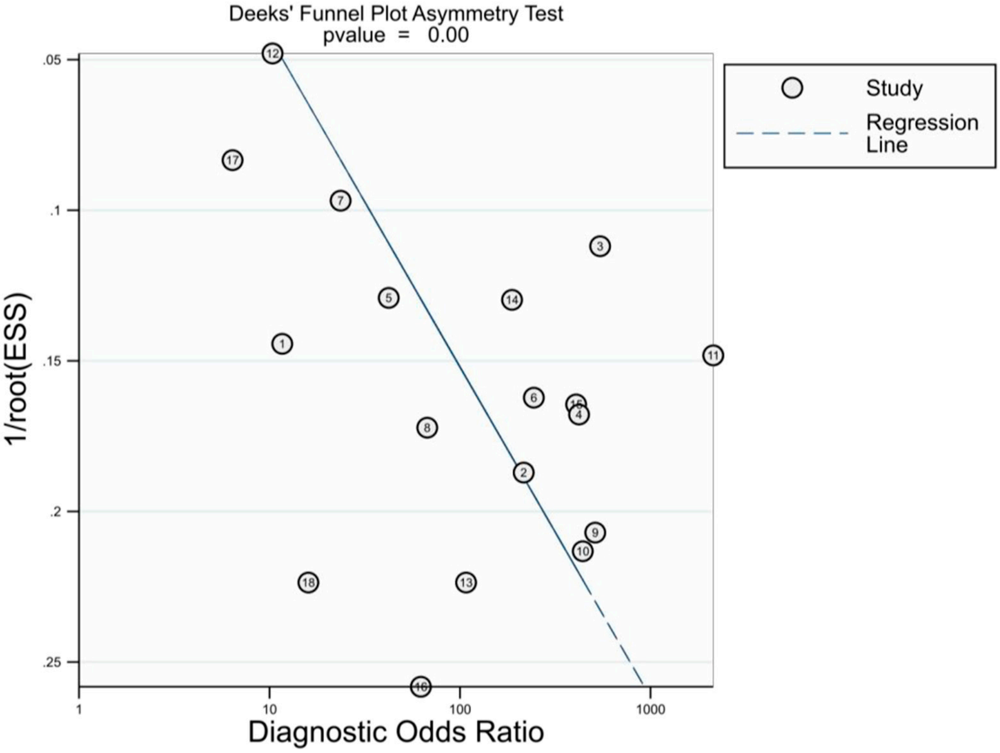

Figure 5. Deeks funnel plot asymmetry test for assessment of publication bias. p-values < 0.05 were considered as significant. ESS: Effective sample size.
